# Controlling the Structures, Flexibility, Conductivity Stability of Three-Dimensional Conductive Networks of Silver Nanoparticles/Carbon-Based Nanomaterials with Nanodispersion and their Application in Wearable Electronic Sensors

**DOI:** 10.3390/nano10051009

**Published:** 2020-05-25

**Authors:** Chih-Wei Chiu, Jia-Wun Li, Chen-Yang Huang, Shun-Siang Yang, Yu-Chian Soong, Chih-Lung Lin, Jimmy Chi-Min Lee, William Anderson Lee Sanchez, Chih-Chia Cheng, Maw-Cherng Suen

**Affiliations:** 1 Department of Materials Science and Engineering, National Taiwan University of Science and Technology, Taipei 10607, Taiwan; a12352335@yahoo.com.tw (J.-W.L.); d10504015@gapps.ntust.edu.tw (C.-Y.H.); M10504114@mail.ntust.edu.tw (S.-S.Y.); yuchiansoong@gmail.com (Y.-C.S.); jerry@email.ctci.org.tw (C.-L.L.); jimmy@cleaninst.com (J.C.-M.L.); williaxom@gmail.com (W.A.L.S.); 2 Graduate Institute of Applied Science and Technology, National Taiwan University of Science and Technology, Taipei 10607, Taiwan; cccheng@mail.ntust.edu.tw; 3 Department of Fashion Business Administration, LEE-MING Institute of Technology, New Taipei City 24305, Taiwan; sunmc0414@gmail.com

**Keywords:** silver nanoparticles, carbon-based nanomaterials, dispersant, nanohybrid film, electrical conductivity, electrocardiogram

## Abstract

This research has successfully synthesized highly flexible and conductive nanohybrid electrode films. Nanodispersion and stabilization of silver nanoparticles (AgNPs) were achieved via non-covalent adsorption and with an organic polymeric dispersant and inorganic carbon-based nanomaterials—nano-carbon black (CB), carbon nanotubes (CNT), and graphene oxide (GO). The new polymeric dispersant—polyisobutylene-*b*-poly(oxyethylene)-*b*-polyisobutylene (PIB-POE-PIB) triblock copolymer—could stabilize AgNPs. Simultaneously, this stabilization was conducted through the addition of mixed organic/inorganic dispersants based on zero- (0D), one- (1D), and two-dimensional (2D) nanomaterials, namely CB, CNT, and GO. Furthermore, the dispersion solution was evenly coated/mixed onto polymeric substrates, and the products were heated. As a result, highly conductive thin-film materials (with a surface electrical resistance of approximately 10^−2^ Ω/sq) were eventually acquired. The results indicated that 2D carbon-based nanomaterials (GO) could stabilize AgNPs more effectively during their reductNion and, hence, generate particles with the smallest sizes, as the COO^−^ functional groups of GO are evenly distributed. The optimal AgNPs/PIB-POE-PIB/GO ratio was 20:20:1. Furthermore, the flexible electrode layers were successfully manufactured and applied in wearable electronic sensors to generate electrocardiograms (ECGs). ECGs were, thereafter, successfully obtained.

## 1. Introduction

Due to the change in demographics of the global population, the increase in the percentage of elderly people in society may become the most dominant and influential phenomenon in the 21st century [1]. As a result, portable or wearable devices and vital sign measurement techniques have been developed and applied in health monitoring research and healthcare for patients, especially the elderly ones. However, current vital sign measurement devices cause discomfort to long-term users. Hence, various smart textile sensors have been developed [2] and applied in different systems, such as wearable cardiopulmonary monitoring devices [3], wearable electrocardiogram (ECG) recorders [4], and cardiac monitoring systems, that can display the measurements on smartphones [5]. Meanwhile, cardiovascular diseases, including coronary heart diseases and strokes, are one of the most common causes of death worldwide. Patients who have them need long-term and expensive medical treatments [6]. Furthermore, their condition has to be monitored carefully and continuously with the help of ECG devices, whose conductive electrode layers respond to vital signs, transmitting, thereafter, signals to sensors [7]. Commercial electrode layers usually use a conductive Ag/AgCl gel to avoid contact problems between the electrode layers and the skin. This gel, however, often stimulates the skin, causing discomfort [8]. To solve this issue, various studies have been conducted on the electrode layers of ECG devices [9,10]. Conductive materials are mostly metals and inorganic substances, such as gold [11], silver [12], aluminum [13], carbon black (CB) [14], carbon nanotubes (CNT) [15], graphene [16], and others [17]. Furthermore, when one of these raw materials is used in the electrode layers, they become rigid, inflexible, and brittle. As a result, improvements concerning these issues are essential to apply these conductive materials in the electrode layers of wearable ECG devices.

Among these metals and inorganic materials, silver is a precious metal that has a relatively high electrical conductivity and low cost [18,19]. As a result, silver nanoparticles (AgNPs), which are usually synthesized through the reduction reactions of silver salts [20], such as silver nitrate [21] and silver sulfate [22], are widely used in various devices and applications. Furthermore, to avoid a cost increase due to excessive use of silver salts, low-cost inorganic materials are often coated with silver [23,24]. Silver coating techniques, such as thermal decomposition [25], vapor deposition [26], surface chemical reduction [27], and γ-radiation [28], have been, therefore, studied extensively. In these methods, the weak interactions between Ag and inorganic surfaces and the aggregation of AgNPs make the coating on inorganic surfaces impossible [29]. To solve this problem, our research team synthesized a new dispersant that enabled the AgNPs coating on the surfaces of two-dimensional (2D) materials (mica nanosheets), resulting in highly conductive films [30]. Moreover, a variety of allotropes, including graphite, diamond, fullerene, CNT, graphene, and graphene oxide [31], maybe selected for carbon-based nanomaterials, as they exist in various dimensions. In particular, CB (0D nanomaterial), CNT (1D nanomaterial), and graphene (2D nanomaterial) are the typical carbon-based nanomaterials and are electrically conductive, as they have interconnected electrical paths [32]. Hence, they are widely used as nanofillers [33]. Previous research has introduced three materials of different dimensions, namely, graphene, silver nanoparticles, and nanowires, into carbon fiber/epoxy laminates [34]. Physical filler-filler interactions can promote synergy, which probably occurs during electronic transmission and, thereby, influences the conductivity of the materials. Previously, our research team identified that polyisobutylene-*b*-poly(oxyethylene)-*b*-polyisobutylene (PIB-POE-PIB) triblock copolymers could be used as a stabilizer for graphene oxide (GO) and AgNPs [35]. Coating AgNPs onto the surface of graphene oxide can result in a high level of conductivity of 1.4 × 10^−1^ Ω/sq.

However, the sphere-like CB and rod-like CNT, containing a high aspect ratio, may, therefore, provide a high surface area for interaction with AgNPs. To understand the effects of materials of different dimensions on silver coating techniques, this paper adopted PIB-POE-PIB as a stabilizer to coat AgNPs onto the surfaces of carbon-based nanomaterials of different dimensions. Therein, the PIB-POE-PIB triblock copolymers were synthesized from polyisobutylene-g-succinic anhydride (PIB-SA) and poly(oxyethylene)-diamine (POE-2000), whose molecular weight (M_w_) was 2000 g/mol, through continuous amidation and imidation reactions. Afterward, CB, CNT, and GO of different dimensions were coated separately with AgNPs by using PIB-POE-PIB as a stabilizer. The effects of AgNPs coating on the properties of substrates with different geometry were compared. Conductive nanoparticles were, thereafter, coated onto polyimide (PI) thin films to create flexible electrode layers for ECG devices. ECG tests were conducted afterward.

## 2. Materials and Methods

### 2.1. Materials

The carbon black (CB), carbon nanotube (CNT), and graphene oxide (GO) used in this study were obtained from Enerage Inc., Yilan, Taiwan. Silver nitrate (purity 99.9%) was obtained from Sigma-Aldrich Chemical Co., St. Louis, MO, USA, and *N*,*N*-dimethylformamide (DMF) was obtained from TEDIA Company Inc., Fairfield, OH, USA. Polyisobutylene-g-succinic anhydride (PIB-SA, M_w_ = 1335) was purchased from Chevron Corp., San Ramon, USA. Poly(oxyethylene)-diamine (POE-2000, M_w_ = 2000), with the designated trade name of Jeffamine^®^ ED2003, was obtained from Huntsman Chemical Co., Los Angeles, CA, USA. Polyimide (PI) film (the degradation temperature about 550 °C, and the thickness was 25 µm) was obtained from UNION CHEMICAL IND. CO. LTD., Taipei, Taiwan.

### 2.2. Synthesis of Organic Dispersant of Polyisobutylene-b-Poly(oxyethylene)-b-Polyisobutylene (PIB-POE-PIB) Triblock Copolymers

According to previous research [35], PIB-POE-PIB was synthesized through the amidation and imidation reactions of PIB-SA and POE-2000 solutions with a molar ratio of 1:2 of organic solvent—tetrahydrofuran (THF). First, 0.005 mol of POE-2000 and 30 mL of THF were poured into a 100-mL three-neck round-bottom flask, which was equipped with a magnetic stirrer and a temperature sensor. Afterward, 0.01 mol of PIB-SA (26.8 g) and 20 mL of THF were added to the flask while the mixture was vigorously stirred. The resulted solution was, thereafter, placed at room temperature (25 °C) for 3 h for continuous ring-opening reactions, heated to 180 °C, and rested for 3 h for continuous ring-closing reactions. Finally, the mixture was analyzed using a Fourier transform infrared (FTIR) Spectrometer.

### 2.3. Preparation of AgNPs/PIB-POE-PIB/carbon-based Nanomaterial Hybrids

After the PIB-POE-PIB dispersion in solutions of carbon-based nanomaterials of different dimensions, silver nitrate (AgNO_3_) was reduced to prepare a stable solution of AgNPs. Afterward, 0.2 g of AgNO_3_ was added to 20 mL of deionized (DI) water, followed by the slow addition of the solutions prepared in the previous step. The weight ratios of AgNO_3_, PIB-POE-PIB, carbon-based nanomaterial (CB, CNT, and CO) were 5:5:1, 10:10:1, and 20:20:1, respectively. The mixtures were continuously stirred for a few hours at 80 °C. During this process, the color of the mixtures changed from light yellow to dark yellow, indicating that AgNPs reduced from Ag^+^ to Ag^0^ due to the mixing of AgNO_3_, PIB-POE-PIB, and carbon-based nanomaterials. As a result, homogeneous suspensions of AgNPs were obtained, and their particle size was observed using a transmission electron microscope.

### 2.4. Synthesis of PI Electrode Layers Using Hybrids of AgNPs/PIB-POE-PIB/Carbon-Based Nanomaterial

First, solutions of AgNPs/PIB-POE-PIB/carbon-based nanomaterial were coated on flexible PI films with a thickness of 250 nm by an automatic coating machine. Afterward, the flexible thin-film PI electrode layers were obtained and sintered at approximately 360 °C using a heater. They were cut into two identical pieces (3 cm × 8 cm), resulting in electrode patches.

### 2.5. Characterization and Instruments

An FTIR spectrometer manufactured by Digilab (Hopkinton, MA, USA), model FTS-1000, was used in a series of tests, ranging from 400 to 4000 cm^−1^, in which samples were scanned 16 times on average to obtain the FTIR spectra. Meanwhile, the ultraviolet-visible light (UV-vis) spectra were obtained using a V-630 mode Spectrophotometer (JASCO Corporation, Kyoto, Japan). A nanohybrid solution, whose mass concentration was 1 wt.%, was synthesized, diluted, and tested to analyze the adsorption of specific wavelengths in the UV-vis spectra and, therefore, to examine the reduction reactions of silver. Gel permeation chromatography (GPC, Waters Instruments Model ACQUITY APC, Taipei, Taiwan) was used to determine the molecular weights of polymers by conducting tests at 45 °C. THF was utilized as the solvent and allowed to flow at 0.8 mL/min. A transmission electron microscope (TEM, Zeiss EM 902A, Oberkochen, Germany) was utilized to observe the size of the AgNPs. To obtain this, the measurement sample solutions with mass concentrations of 1 wt.% were prepared. The solutions were, thereafter, deposited onto carbon-coated copper meshes, vacuumed until they were dry, and their residues were examined using a TEM. All silver-containing conductive thin films were tested by a four-point sheet resistance meter (MCP-T610, Keithley Instruments, Inc., Cleveland, OH, USA) to evaluate their electrical conductivity. The sheet resistance of the film was determined by the four-point probe method using co-linear and four equally-spaced probes to measure electrical contact with the material. In addition, thermogravimetric analysis (TGA) was performed by using TA instruments, Q-500 (New Castle, DE, USA). Weight loss curves were obtained by considering samples of 5–8 mg and heating them to 720 °C in nitrogen. Furthermore, a high-resolution field-emission scanning electron microscope (FESEM), model JSM-6500F (JEOL, Tokyo, Japan), was also utilized to obtain images of test samples, which were prepared after dropping previous samples onto clean glass surfaces and drying them for 2 h at 80 °C, fixing them to a conductive carbon paste and coating them with a thin film of platinum (Pt). Finally, two pieces of PI electrode patches were fixed onto an elastic band with buttons to create heart rate monitoring belts, which could be attached to the skin. These belts were integrated with ECG devices from Singular Wings Medical Co., Ltd., Hsinchu, Taiwan, to measure the waveform changes of P-, Q-, R-, S-, and T-waves at rest and during exercise, such as raising hands, squatting, jogging, and cycling.

## 3. Results and Discussion

### 3.1. Synthesis of the PIB–POE–PIB Triblock Copolymer for Use as an Organic Dispersant

**Figure 1:**
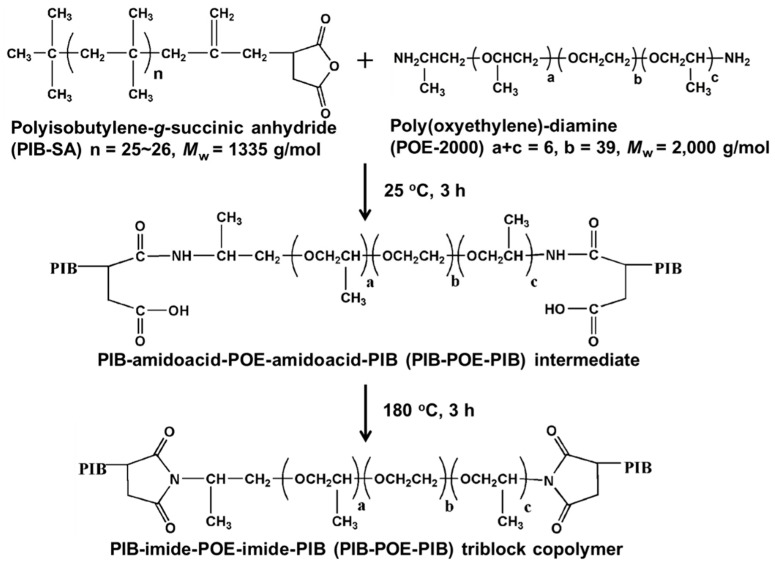
Synthesis of polyisobutylene-*b*-poly(oxyethylene)-*b*-polyisobutylene (PIB-POE-PIB) triblock structures (polymeric dispersants) from polyisobutylene-*g*-succinic anhydride (PIB-SA) and poly(oxyethylene)-diamine (POE-2000) via amidation and then imidation reactions.

As shown in Figure 1, PIB-SA was composed of hydrophobic groups, while polyethylene glycol in POE-2000 was composed of hydrophilic groups. These two materials were subjected to cyclic anhydride ring-opening reactions at 25 °C and, afterward, three-hour amidation, in which hydroxyl groups were replaced by amine groups (–NH_2_). As a result, an organic dispersant (PIB-amide-PIB), composed of polar and nonpolar groups, was synthesized. To ensure the stability of the dispersant, amide groups were, further, subject to imidation at 180 °C for 3 h, resulting in the organic dispersant used in this research. The product was preliminarily analyzed using GPC to determine its molecular weight. Figure S1 (Supplementary Materials) shows the GPC curves of the raw materials (PIB-SA and POE-2000) and PIB-POE-PIB, while the GPC results are summarized in Supplementary Materials (Table S1). The average molar mass (Mn) of PIB-SA, EN2003, and PIB-POE-PIB was 1785, 2841, and 4696 g/mol, respectively. The molecular weights of PIB-SA and EN2003 were higher than the theoretical value (1335 and 2003 g/mol, correspondingly), probably due to experimental errors caused by the relatively small molecular weights of these raw materials. Meanwhile, the theoretical molecular weight of PIB-POE-PIB (4673 g/mol) was similar to the experimental result (4696 g/mol), suggesting that the raw materials reacted, producing the PIB-POE-PIB. The FTIR spectra of PIB-SA, PIB-amide-PIB, and PIB-POE-PIB are shown in Supplementary Materials (Figure S2). For PIB-SA, peaks, indicating stretching vibrations of anhydride (C=O), occurred in 1780 and 1710 cm^−1^. After amidation, these peaks disappeared from the FTIR spectrum of PIB-amide-PIB; however, typical adsorption peaks, indicating the presence of amide (–CONH), occurred at 1640 and 1570 cm^−1^, suggesting the end of the amidation of PIB-SA and POE-2000. After the ring-closing reactions of PIB-POE-PIB intermediate at 180 °C for 3 h, peaks, indicating –NH stretching vibrations, disappeared after 1570 cm^−1^. Furthermore, peaks, indicating the stretching vibrations of C=O in imide, occurred at 1710 and 1650 cm^−1^, corroborating that PIB-POE-PIB was formed due to ring-closing reactions of PIB-amido acid-PIB. Furthermore, in this study, different solvents were tested to evaluate their compatibility with PIB-POE-PIB and to choose the most suitable for a silver reduction. The results are as shown in Supplementary Materials (Figure S3), and the solubility data are summarized in Supplementary Materials (Table S2). As a result, PIB-SA was not soluble in DI water; however, after PIB-SA reacted with POE-2000, which had high solubility considering all solvents tested, the resulting PIB-POE-PIB solution had satisfactory solubility in most organic solvents, while it partially dissolved in DI water. As the water was used to dissolve AgNO_3_ during the reduction of silver, an improvement in PIB-POE-PIB solubility in DI water might stabilize the silver reduction reactions. Finally, the dispersibility of nano-CB in DI water, DMF, and DMF containing PIB-POE-PIB was analyzed. The nano-CB showed high levels of aggregation in pure solvents. After PIB-POE-PIB had been dissolved in DMF, a homogeneous nano-CB solution was obtained. Hence, PIB-POE-PIB was the most appropriate dispersant for silver reduction.

### 3.2. Preparation of Colloidal AgNPs Dispersed in PIB-POE-PIB/Carbon-Based Nanohybrids as Organic/Inorganic Dispersants

Figure S4a (Supplementary Materials) shows the UV spectra of the products from the reduction of AgNO_3_ due to PIB-POE-PIB, at different weight ratios of AgNO_3_ to PIB-POE-PIB (1:1, 5:1, and 1:5). The peaks were located at approximately 410 nm in all scenarios. Hence, the UV spectra demonstrated that the products of all samples were nanoparticles, corresponding to the localized surface plasmonic effects of AgNPs [36,37]. To obtain the optimal ratio, TEM was adopted to measure the diameters of AgNPs. As illustrated in Figure S4b, when the weight ratio of AgNO_3_ to PIB-POE-PIB was 1:1, a more uniform distribution of the diameters of AgNPs was obtained. In the situation in which the ratio was 1:5, the diameters of AgNPs were higher by approximately one time. As the diameters and uniformity of this material could affect the electrical conductivity of AgNPs/PIB-POE-PIB, a 1:1 weight ratio of AgNO_3_ to PIB-POE-PIB had been selected. The sheet resistance of the AgNPs/PIB-POE-PIB powder reduced at this ratio was measured using a four-point sheet resistance meter (Table 1). The result suggested that the AgNPs/PIB-POE-PIB powder that was reduced at a 1:1 ratio had satisfactory electrical conductivity, and therefore, the same ratio was set 1:1 for subsequent tests, as the resulting product was electrically conductive, and a relatively uniform layer of AgNPs could be obtained.

**Figure 2:**
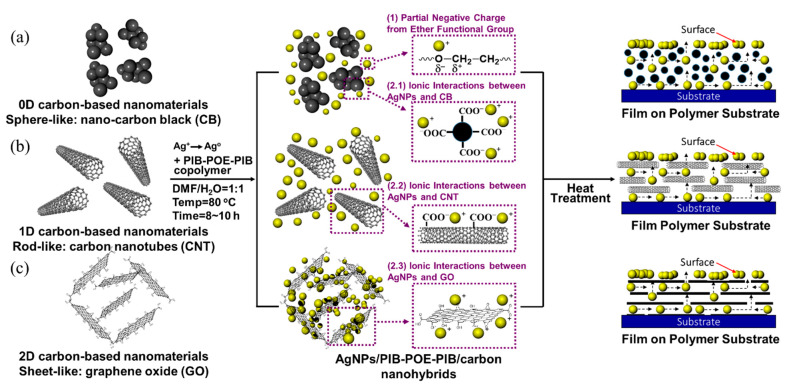
Adsorption mechanisms for dispersion and stabilization of the three silver nanoparticles (AgNPs)/PIB-POE-PIB/carbon-based nanomaterial hybrids according to various types of intermolecular forces. (**a**) AgNPs/PIB-POE-PIB/nano-carbon black (CB), (**b**) AgNPs/PIB-POE-PIB/carbon nanotubes (CNT), and (**c**) AgNPs/PIB-POE-PIB/graphene oxide GO.

As illustrated in Figure 2, dipole-dipole adsorption occurred between O^−^ of POE-2000 chains of PIB-POE-PIB and Ag^+^ of AgNO_3_, whereas dispersion of carbon-based nanomaterials was promoted by the hydrophobic long chains of PIB-SA chains. As a result, both AgNO_3_ and carbon-based nanomaterials could be effectively dispersed in PIB-POE-PIB solutions. Hence, in a CB system, Figure 2a, the COO^−^ functional groups on the CB surfaces could form ionic bonds with Ag^+^ of AgNO_3_. Due to ionic bond interactions and van der Waals forces, AgNO_3_ could be distributed on the surfaces of CB, resulting in the reduction of the component. The COO^−^ functional groups were placed on the surfaces of CB, comprising the CB system, which could be compared with the CNT and GO systems (Figure 2b,c). For all systems, silver networks were formed on their surfaces after heating them due to the low melting point of silver nanoparticles [35].

**Figure 3:**
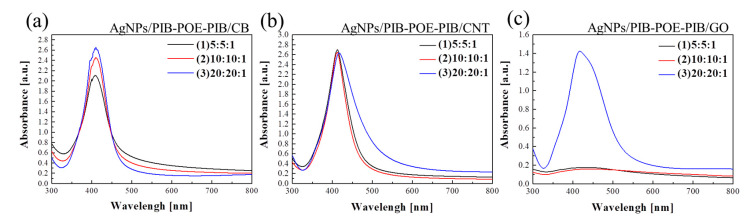
UV-vis absorption spectra of AgNPs/PIB-POE-PIB/carbon-based nanomaterial hybrids of different weight ratios. (**a**) AgNPs/PIB-POE-PIB/CB, (**b**) AgNPs/PIB-POE-PIB/CNT, and (**c**) AgNPs/PIB-POE-PIB/GO.

**Figure 4:**
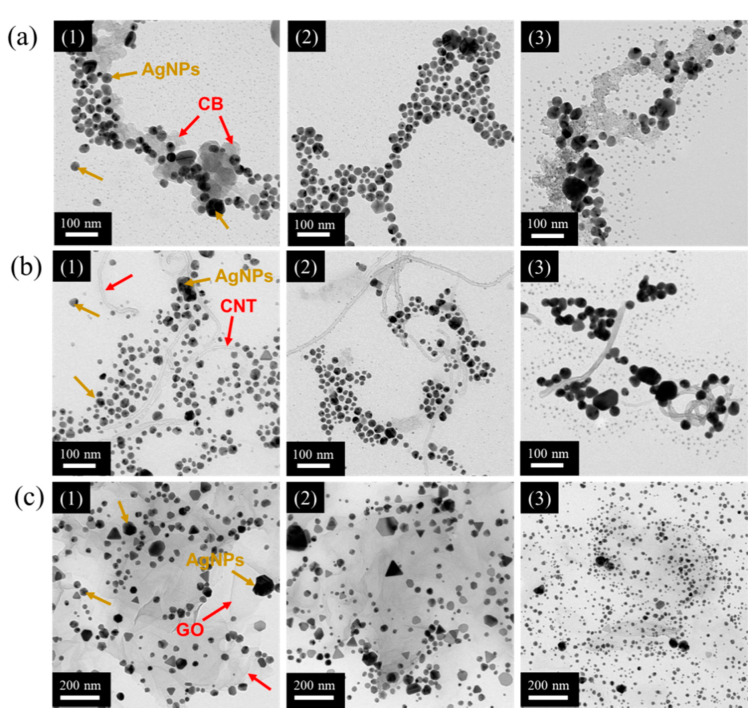
Transmission electron microscope (TEM) images of colloidal AgNPs in complexes with different weight ratios of AgNO3, PIB-POE-PIB, and carbon-based nanomaterials. (**a**) AgNO3/PIB-POE-PIB/CB, (**b**) AgNO3/PIB-POE-PIB/CNT, and (**c**) AgNO3/PIB-POE-PIB/GO reductions with weight ratios of (1) 5:5:1, (2) 10:10:1, and (3) 20:20:1.

Meanwhile, the UV spectra of the silver reduction products from AgNO_3_/PIB-POE-PIB/carbon-based nanomaterial with ratios of 5:5:1, 10:10:1, and 20:20:1 (Figure 3) revealed that peaks for all samples were located at approximately 414–416 nm. As a result, for all cases, AgNO_3_ was successfully reduced to AgNPs. TEM was, thereafter, employed to measure the sizes of AgNPs for all samples (Figure 4a–c). After the AgNO_3_ reduction (CB, CNT, and GO), AgNPs could be distributed on the surfaces of carbon-based nanomaterials for all systems. Subsequently, TEM images were used to calculate the average particle sizes (Table 1). The average particle size of AgNPs was 16.7–32.8 nm for all samples, proving that PIB-POE-PIB could effectively stabilize the reduction of AgNPs and suggesting that, for both the CB and CNT systems, a solution composed of AgNO_3_, PIB-POE-PIB, carbon-based nanomaterial, whose ratio was 10:10:1, could form smaller and more uniform particles of AgNPs. As a result, these particles could adhere to the surfaces of CB, CNT, and polymer chains. For the GO system, a ratio of 20:20:1 could generate the smallest particles of AgNPs among all systems covered in this work, probably as GO is a 2D carbon-based nanomaterial. During the reduction of AgNPs, 2D GO resulted in a uniform distribution of COO^−^ functional groups. Due to the interactions of the more uniformly distributed ionic bonds and van der Waals forces, the reduction of AgNPs became more stable, and therefore, small particles of AgNPs were synthesized. Due to the small particle size, the surface area of the AgNPs increased, and therefore, the AgNPs material could interact more effectively. As a result, the highest conductivity of the solution (AgNO_3_/PIB-POE-PIB/GO) could be achieved at a ratio of 20:20:1.

### 3.3. Preparation of Highly Conductive Films

Degradation of hybrid thin films composed of AgNPs/PIB-POE-PIB/carbon-based nanomaterial (CB, CNT, and GO systems) was analyzed at high temperatures using TGA (Supplementary Materials, Figure S5). The results indicated that the degradation of polymer chains of PIB-POE-PIB occurred at approximately 350–360 °C for all samples. Furthermore, staged high-temperature sintering of solutions composed of AgNPs/PIB-POE-PIB/carbon-based nanomaterial was conducted to observe the changes on the thin film surfaces (Supplementary Materials, Figure S6). For a CB system, after sintering at 80 °C for 30 min, the thin film had a black color similar to that of the nanohybrid solution. This color, however, changed at 180 °C due to the aggregation of AgNPs, which were drawn to the surface because of their low melting point. As the temperature was raised until 300–360 °C, a whitish nanohybrid thin-film started to form on the surface, as the organic dispersant (PIB-POE-PIB) was gradually degraded. As a result, a silver network was formed on the thin film surface.

**Figure 5:**
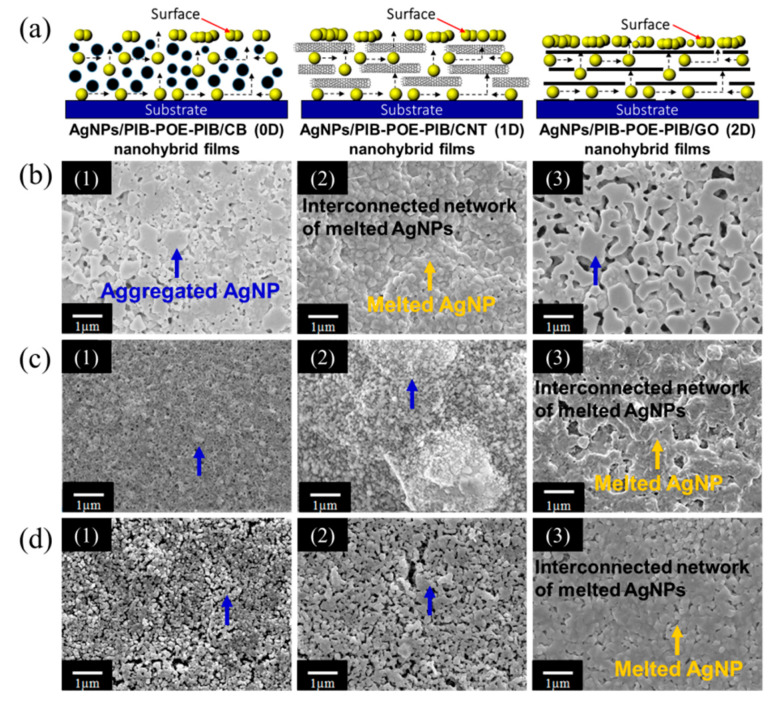
(**a**) Schematic diagram showing the migration of AgNPs to the film surface with the three AgNPs/PIB-POE-PIB/carbon-based nanomaterial hybrids. (1) AgNPs/PIB-POE-PIB/CB, (2) AgNPs/PIB-POE-PIB/CNT, and (3) AgNPs/PIB-POE-PIB/GO. FE-SEM image during the melting of AgNPs on the surfaces of the flexible polyimide substrates. Formation of whitish thin films and conductivity value can be analyzed using the FE-SEM images taken when AgNPs/PIB-POE-PIB/carbon-based nanomaterials thin films with different weight ratios were heated at 360 °C. (**b**) AgNO_3_/PIB-POE-PIB/CB, (**c**) AgNO_3_/PIB-POE-PIB/CNT, and (**d**) AgNO_3_/PIB-POE-PIB/GO reductions at weight ratios of (1) 5:5:1, (2) 10:10:1, and (3) 20:20:1.

Meanwhile, the AgNPs/PIB-POE-PIB/CNT thin film was still black at 300 °C, and a whitish nanohybrid thin-film appeared only at 360 °C. Finally, the AgNPs/PIB-POE-PIB/GO thin film darkened after 300 °C, also becoming a whitish nanohybrid thin film at 360 °C. The systems showed different results, especially as the space obstruction of 0D, 1D, and 2D materials differed. On comparing the AgNPs of the 1D CNT system, those in the CB (0D) system were drawn to the surface more easily during melting. In the GO (2D) system, the space obstruction was significant and needed to melt and draw these particles. SEM was, thereafter, employed to observe the surface morphology of samples that were prepared with different weight ratios of carbon-based nanomaterials, after the synthetization at 360 °C (Figure 5). The results demonstrated that high-temperature sintering of AgNPs/PIB-POE-PIB/carbon-based nanomaterial resulted in compact silver network structures, which were different for samples with different weight ratios of carbon-based nanomaterials. The sheet resistance of the sintered samples was measured using a four-point sheet resistance meter (Table 1). For the CB systems, when the AgNPs/PIB-POE-PIB/CB ratio was 10:10:1, the optimal conductivity was achieved, as the particle size of AgNPs was small. When a smaller was achieved, these particles could be more uniformly distributed on carbon-based nanomaterials; hence, when 0D materials were used, high-temperature sintering might result in more regular and continuous silver networks, which were generated after melting them. Meanwhile, for the CNT and GO systems, ratios of 20:20:1 resulted in the highest conductivity, probably due to the space obstruction of 1D and 2D materials that caused an increase in the number of AgNPs required to form the relatively compact silver layers in the silver networks. In general, the conductivity of the sample, composed of AgNPs, PIB-POE-PIB, and GO, and whose ratio was 20:20:1, was higher than that of the samples composed of CB and CNT. Due to the more uniform distribution of COO^−^ functional groups in 2D GO, the smallest AgNPs could be obtained during the reduction of AgNPs. Furthermore, as the number of AgNPs increased, the adherence to the surfaces of GO improved. Moreover, SEM images indicated that the sample whose ratio was 20:20:1 had interconnected conductive structures and the most compact silver network structures, leading to the highest conductivity among the systems. Finally, the conductivity of the AgNPs/PIB-POE-PIB/carbon-based nanomaterial thin films was evaluated when they were connected to a circuit (Supplementary Materials, Figure S7). The thin films were subjected to flexural fatigue tests for 5000 times. The obtained surface resistance (10^−2^ Ω/sq) was approximately constant during the experiment (Table 1).

### 3.4. Preparation of Wearable Electronic Sensors and ECG Monitoring at Rest and during Exercise

**Figure 6:**
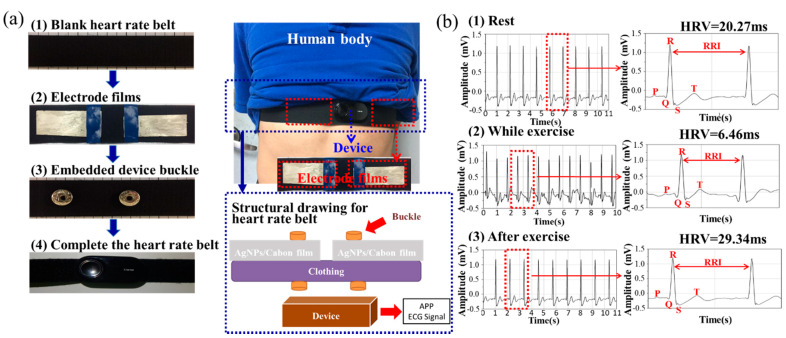
Electrodes of electrocardiograms (ECG) sensors were made using AgNPs/PIB-POE-PIB/GO hybrid thin films, and ECG monitoring was conducted. (**a**) Assembly of the heart rate monitoring belt using AgNPs/PIB-POE-PIB/GO hybrid thin films, and (**b**) Complete and satisfactory signals for QRS-complexes and P- and T-waves in ECGs when the subject was at rest, exercising, and after the exercise.

Electrode layers for ECG sensors and ECG devices (Figure 6) were prepared to measure the surface electrical resistance of nanohybrid electrode patches (Table 1). The AgNPs/PIB-POE-PIB/GO hybrid thin-film before sintering had the highest conductivity, possibly as the grafting rate of the reduced AgNPs onto the surfaces of GO (2D substrates) were influenced by the interactions between them. As a result, relatively discontinuous AgNPs were formed. This issue was solved when 0D, 1D, and 2D carbon-based materials were used; however, the 3D structural obstruction of 2D materials adversely affected the formation of continuous AgNPs. Hence, GO, as it is a 2D plate material, resulted in the product with the highest conductivity due to unprocessed AgNPs that did not influence the system, and to the 3D structural obstruction on AgNPs/PIB-POE-PIB/GO. When the electrode patches of ECG sensors have a resistance smaller than 1.2 × 10^1^ Ω/sq–3.5 × 10^−2^ Ω/sq [38,39], heart rates can be successfully measured. In this research, the synthesized electrode patches were connected to sensor devices and attached to the chest skin of the test subject. The ECG utilizes small electrical changes, which are detected, amplified (to avoid inaccurate measurement), and stored by ECG devices, caused by depolarization of the cardiac muscle during each cardiac cycle. In this study, ECGs at rest, during exercise (cycling), and after exercise were obtained, and signals recorded after 10 seconds from the beginning of the exercise were compared to those at rest and after exercise. The intervals between signals were reduced during exercise, indicating that the heart rate of the subject increased. Afterward, the R-R intervals (RRI) of the ECG signals were amplified to complete the analysis. The results revealed that distinct QRS-complexes and P- and T-waves were present in the ECGs regardless of the state of the subject. Furthermore, the standard deviations of all RR intervals (SDNN) of the ECG signals were calculated to obtain the heart rate variability (HRV) from the RRI. HRV represents the total autonomic activity; therefore, high values of HRV indicate good autonomic adjustment. The results showed that the heart rate monitoring belts developed in this research could successfully measure at different states (at rest, during exercise, or after exercise) the HRV values (20.27, 6.46, and 29.34 ms, respectively). The results indicated that the HRV value during exercise was lower; therefore, the current intensity of the exercise was relatively low to the subject and could be increased further. Although the RRI value measured by the ECG chest strap was not as accurate as those obtained using medical instruments, it was more accurate than those measured by other ECG wrist or finger devices, for example, photoplethysmography (PPG). In hospitals, at least three electrodes are needed to mark P-, T-, and U-waves and to create an ECG. Hence, although the results were slightly inaccurate, the combination of heart rate monitoring chest straps and mobile applications (APPs) was a simple and inexpensive alternative to analyze the HRV and to monitor the health status of a subject. Finally, ECG devices using CNT and CB were also validated (Supplementary Materials, Figures S8 and S9). Furthermore, a video showing the flexibility, conductivity stability, and high sensitivity of the ECG electrodes using the nanohybrid film is provided in the Supplementary Material (Video S1).

## 4. Conclusions

In recent years, people have shown more interest in doing exercises; however, the changes in demographics indicate that the percentage of elderly people in society will increase. As a result, vital sign measuring devices have become more popular. Among these devices, small heart rate monitoring devices have become the most popular ones due to their convenience. In this paper, materials to produce electrodes for the sensors used in this device were successfully synthesized using AgNPs and carbon-based nanomaterials. Moreover, a heart rate monitoring belt was created, and vital signs were successfully measured. Highly stable AgNPs dispersants used in this study were obtained from an organic polymeric dispersant and inorganic carbon-based nanomaterials. They could effectively limit the size of the AgNPs to 16.7–32.8 nm. When PIB-POE-PIB and carbon-based nanomaterials were used as dispersants for AgNPs, the solution composed of AgNPs, PIB-POE-PIB, and GO, whose ratio was 20:20:1, led to a relatively satisfactory dispersion of AgNPs. In addition, the resulting nanohybrid solution had the highest conductivity. Furthermore, the complete degradation of PIB-POE-PIB at 360 °C resulted in the formation of electrically conductive network structures on the surfaces of the nanohybrids by AgNPs. SEM images also revealed that interconnected conductive structures were created due to the most compact silver network structures formed at the mentioned ratio, probably as the 2D structures of GO led to a more uniform distribution of the CCO^−^ functional groups. The surface resistance of this highly conductive flexible nanohybrid thin film was approximately 10^−2^ Ω/sq. Finally, the feasibility of these flexible electrode layers in wearable electronic ECG devices was validated.

## Figures and Tables

**Table 1:** Particle size, sheet resistance, and mechanical stability of AgNPs/PIB-POE-PIB/carbon-based nanomaterials hybrids.

Sample	Weight Ratios	Average Ag Particle Size (nm) ^a^	Sheet Resistance (Ω/sq) ^b^
CB	--	--	2.3 × 10^1^
CNT	--	--	9.2 × 10^2^
GO	--	--	8.6 × 10^6^
AgNPs/PIB-POE-PIB	1:1	29.9	4.5 × 10^4^
AgNPs/PIB-POE-PIB/CB	5:5:1	26.2	2.8 × 10^−1^
	10:10:1	25.4	2.0 × 10^−2^ (4.0 × 10^−^^1^) ^c^
	20:20:1	28.4	4.4 × 10^−1^
AgNPs/PIB-POE-PIB/CNT	5:5:1	29.8	5.2 × 10^0^
	10:10:1	28.1	6.5 × 10^0^
	20:20:1	32.8	9.4 × 10^−2^ (1.8 × 10^−^^1^) ^c^
AgNPs/PIB-POE-PIB/GO	5:5:1	--	3.4 × 10^−1^
	10:10:1	--	1.5 × 10^−1^
	20:20:1	16.7	1.2 × 10^−2^ (2.4 × 10^−2^) ^c^

^a^ Average particle size of the silver nanoparticles (AgNPs) measured by transmission electron microscope (TEM). ^b^ Solution was coated on polyimide substrates, and the sheet resistance was measured using a four-point sheet resistance meter. ^c^ The mechanical stability of the flexible electrodes was tested by conducting flexural fatigue tests 5000 times.

## Supplementary Materials

The following are available online at https://www.mdpi.com/2079-4991/10/5/1009/s1, Figure S1: GPC curves for PIB-SA, POE-2000, and PIB-POE-PIB triblock copolymer. Figure S2: FT-IR spectra of PIB-SA, PIB-POE-PIB intermediate, and PIB-POE-PIB triblock copolymer. Figure S3: (a) PIB-POE-PIB triblock copolymer in different solvents: (1) DI Water, (2) DMF, (3) acetone, (4) ethanol, (5) THF, and (6) toluene. (b) Carbon black dispersed in (1) DI water, (2) DMF without PIB-POE-PIB, and (3) DMF with PIB-POE-PIB. Figure S4: (a) UV-vis absorption spectra. (b) TEM images of colloidal AgNP in DI water containing AgNO_3_ and PIB-POE-PIB at weight ratios: (1) 1:1, (2) 5:1, and (3) 1:5. Figure S5: TGA plots of AgNPs/PIB-POE-PIB/carbon-based nanomaterials hybrids with different weight ratios. (a) AgNPs/PIB-POE-PIB/CB, (b) AgNPs/PIB-POE-PIB/CNT, and (c) AgNPs/PIB-POE-PIB/GO. Figure S6: Preparation of highly electrically conductive films. Photos of AgNP/PIB-POE-PIB/carbon-based nanomaterials hybrid films with a weight ratio of 20:20:1 heated at 80 °C, 180 °C, 300 °C, and 360 °C for 30 min. (a) AgNP/PIB-POE-PIB/CB, (b) AgNP/PIB-POE-PIB/CNT, and (c) AgNP/PIB-POE-PIB/GO. Figure S7: (a) A circuit connected using (1) a non-conductive PI film and (2) an AgNP/PIB-POE-PIB/GO thin film. The latter is shown to be conductive. (b) After the thin film was subjected to flexural fatigue tests 5000 times, its sheet resistance was constant and approximately 10^−2^ Ω/sq. (c) The result showed that the sheet resistance of the thin film was maintained by at least 10^−2^ Ω/sq after a durability test. (d) The cross-sectional and (e) the surface view of the nanohybrid film after flexural fatigue tests 5000 times. Figure S8: (a) Optical images of heart rate monitor device featuring AgNP/PIB-POE-PIB/CNT hybrid electrodes. (b) When the body was at rest and during exercise, complete and satisfactory signals for QRS-complexes and P- and T-waves were acquired from the obtained ECGs. Figure S9: (a) Optical images of heart rate monitor device featuring AgNP/PIB-POE-PIB/CB hybrid electrodes. (b) Complete and satisfactory signals for QRS-complexes and P- and T-waves were acquired from the ECGs when the subject was at rest and during exercise. Table S1: GPC results of PIB-SA, POE-2000, and PIB-POE-PIB triblock copolymer. Table S2: The solubility of PIB-POE-PIB triblock copolymer in various solvents. Video S1: A video showing a demonstration of the LED bulbs illuminated using the high conductive films and a video depicting a highly sensitive ECG measurement by high conductive films as flexible electrodes under dynamic activity are provided.
